# Energy recovery of waste plastics into diesel fuel with ethanol and ethoxy ethyl acetate additives on circular economy strategy

**DOI:** 10.1038/s41598-022-09148-2

**Published:** 2022-03-29

**Authors:** Sambandam Padmanabhan, K. Giridharan, Balasubramaniam Stalin, Subramanian Kumaran, V. Kavimani, N. Nagaprasad, Leta Tesfaye Jule, Ramaswamy Krishnaraj

**Affiliations:** 1 School of Mechanical and Construction, Vel Tech Rangarajan Dr. Sagunthala R&D Institute of Science and Technology, Chennai, Tamil Nadu 600 062 India; 2 Department of Mechanical Engineering, Easwari Engineering College, Chennai, Tamil Nadu 600 089 India; 3 Department of Mechanical Engineering, Anna University, Regional Campus Madurai, Madurai, Tamil Nadu 625 019 India; 4 Centre for Drug Discovery and Development, Sathyabama Institue of Science and Technology, Chennai, Tamil Nadu India; 5 Department of Mechanical Engineering, Karpagam Academy of Higher Education, Coimbatore, Tamil Nadu 641 021 India; 6 Department of Mechanical Engineering, ULTRA College of Engineering and Technology, Madurai, Tamil Nadu 625 104 India; 7 Centre for Excellence-Indigenous Knowledge, Innovative Technology Transfer and Entrepreneurship, Dambi Dollo University, Dambi Dollo, Ethiopia; 8 Department of Physics, College of Natural and Computational Science, Dambi Dollo University, Dambi Dollo, Ethiopia; 9 Department of Mechanical Engineering, Dambi Dollo University, Dambi Dollo, Ethiopia

**Keywords:** Biochemistry, Biogeochemistry, Environmental sciences, Environmental social sciences, Energy science and technology, Engineering

## Abstract

The widespread use of plastic goods creates huge disposal issues and environmental concerns. Increasing emphasis has been paid to the notion of a circular economy, which might have a significant impact on the demand for plastic raw materials. Post-consumer plastics recycling is a major focus of the nation’s circular economy. This study focuses on energy recovery from waste plastics as an alternative fuel source to meet the circular economy demand. Waste plastic fuel produced through pyrolysis has been claimed to be utilized as a substituted fuel. This work focuses to determine the performance and emission standards of Waste Plastic Fuel (WPF) generated from the pyrolysis of High-Density Polyethylene (HDPE) in a single-cylinder Direct Injection Diesel Engine (DIDE). Three different ratios of WPF were combined with 10% ethanol and 10% ethoxy ethyl acetate as an oxygenated additive to create quaternary fuel blends. The ethanol has a low viscosity, a high oxygen content, a high hydrogen-to-carbon ratio as favourable properties, the quaternary fuel results the improved brake thermal efficiency, fuel consumption and reduced emissions. The blend WEE20 exhibits 4.7% higher brake thermal efficiency, and 7.8% reduced fuel consumption compared to the diesel. The quaternary fuel blends demonstrated decreased carbon monoxide of 3.7 to 13.4% and reduced hydrocarbons of 2 to 16% under different load conditions.

## Introduction

Plastic consumption is anticipated to double in the next two decades, after doubling in the prior five decades. To reduce environmental difficulties, the polymer industry must focus on recovering value-added goods rather than single-purpose plastics. The circular economy concept has gained momentum, and it includes an aspiring strategy to increase post-consumer plastic recycling ideas. Reduced consumption, increased life expectancy, recycling, and post-consumer energy recovery are all recommended strategies for reducing pollution caused by plastics. Mechanical recycling is crucial to the circular economy, yet impediments such as incompatible mixing, reduced mechanical properties, and strengthening additives impede the circular economy. Thermal recycling or combustion are used to dispose of plastics are gained more focus. Waste plastic disposal presents a significant opportunity for energy recovery. Hydrocarbons are present in plastic, are a great source of fuel since they burn cleanly. In addition to being ecologically friendly and cost-effective, pyrolysis is a technology for recovering energy from waste plastic that used to reuse plastic waste as a source of energy for fuel production while also being environmentally friendly and cost-effective^1^.

The use of diesel fuels is widespread in many industries, such as the automotive, agricultural, and power generating sectors, which benefit from greater thermal efficiency and superior fuel economy. Looking for alternate sources of fuel is usually a positive experience. Singh et al.^2^ synthesized pure plastic pyrolysis oil without the use of a catalyst and studied the fuel characteristics. The plastic oil blends were tested on the engine, and it was found that utilizing 50% blends resulted in a reduction in efficiency and a minimal rise in emissions. Das et al.^3^ examined waste plastic oil blends produced from the Zeolite-A catalyst. The engine analysis discovered increased brake thermal efficiency up to 20% blends at full load. The exhausting emission is regarded as having a higher value than diesel at greater blend and load ratios. Pyrolysis oils produced from several polymers, including high and low density polyethylene, styrene, and polypropylene were studied by Mangesh et al.^4^. Bukkarapu et al.^5^ studied on the pyrolysis method for converting plastics waste to fuel and reported the use of plastic oil in diesel engines by examining the engine characteristics. Chandran et al.^6^ reported the chemical and physical characteristics of different waste plastic oil mixes with diesel-like polymers and tire oil blends, distilled and desulphurized waste plastic oil.

Fayyazbakhsh and Pirouzfar^7^ conducted a review of oxygenated additives for reducing emissions, improving fuel characteristics, and enhancing the performance of DIDEs. They concluded that increasing the alcohol content of diesel enhanced the premixed combustion phase during combustion, thus decreasing emissions. Bridjesh et al.^8^ attempted to substitute diesel with half the quantity of Waste Plastic Oil (WPO) and the additives methoxyethyl acetate and diethyl ether. Sachuthananthan et al.^9^ combined magnesium oxide nanoparticles with plastic pyrolysis oil in various ratios. The investigation was carried out to investigate the influence of compression ignition on the engine's physicochemical characteristics. Mangesh et al.^10^ explored pyrolysis oil hydrogenation as a novel approach for converting unsaturated chemicals to saturated ones. The study examined at the combustion, generation, and emissions of hydrogenated polypropylene pyrolysis oil combined with diesel.

Devaraj et al.^11^ showed that blending diethyl ether increases the cetane number of the plastic blend to greater than that of diesel and WPO not only and decreases smoke emissions. According to Ananthakumar et al.^12^, they tested the performance of a diesel engine using fuel blends of WPO and diethyl ether. The results showed that WPO blends had a lower BTE than diesel and that specific fuel consumption (SFC) had a significantly lower BTE than diesel in all cases. On the other hand, Hydrocarbon (HC) and smoke emissions were similar to diesel. Vijayabalan and Kaimal^13^ examined the diesel engine characteristics, operating on Diethyl Ether (DEE) at a 5–15 percent concentration by volume in WPO. Increases in the DEE of the blends resulted in increased BTE and less fuel consumption. While Carbon monoxide (CO) emissions decreased, unburned hydrocarbon emissions decreased.

Sukjit et al.^14^ conducted experimental research on a diesel engine utilizing plastic oil-caster oil as a fuel in combination with butanol and DEE as additives. When blends of plastic oil, castor oil, and DEE are utilized as fuel, engine emissions are reduced. Ravi and Karthikeyan^15^ suggested that propanol blend with plastic oil was preferable in terms of performance and emission compared to diesel, and in that emissions, norms are reduced. Das et al.^16^ have examined WPO and ethanol for improved performance and lower emissions, and the Taguchi method has been used to optimize the performance and emissions. According to the findings of this research, greater compression ratios and higher loads result in the greatest possible BTE and the lowest possible emissions for 20% ethanol and 20% WPO mixed diesel. An in-depth and comprehensive investigation reveals that waste plastic fuel can be used as a single fuel or combined with base diesel or oxygenated fuel as a binary blend, with or without present engine modifications. It has been observed that several attempts are made to use oxygenated additives. It was discovered that the addition of alcohols to ternary fuel blends avoided the greater kinematic viscosity and density of diesel found in quaternary blends, as well as improved base fuel properties with a significant reduction in emissions^17^.

This study focuses on energy recovery from waste used plastics as the transportation fuel source via circular economy approach. This investigation is to determine the emission and performance characteristics of waste plastic fuel made from the pyrolysis of HDPE in a single-cylinder diesel engine. The combination of alcohol and ethyl oxygenated additives with plastic fuel recovered from waste plastics was not attempted in diesel engine applications. In this investigation, a combination of 10% ethanol and 10% ethoxy ethyl acetate additives were blended with different ratios of waste plastic fuel as a quaternary fuel to achieve better performance in the diesel engine. Hence, this research assesses the emission characteristics and performance of a single-cylinder diesel engine that has been fuelled with waste plastic fuel and oxygenated additives. The results of WPF are evaluated for the sustainability of the environment to meet the circular economy.

## Materials and methods

In the pyrolysis process, waste plastics are converted into alternative energy as fuel for diesel engines. Waste plastic fuel has a wide range of chemical characteristics that vary depending on the grade of plastics utilized and the pyrolysis technique employed. The low calorific value and high viscosity of the waste plastic fuel are the two most significant drawbacks of utilizing plastic fuel as a diesel engine. HDPE is known for its structure as a linear long-chain polymer with a considerable degree of crystallinity and little branching that ends up with high endurance characteristics. According to forecasts, global demand for HDPE will reach roughly 95 billion tonnes by 2025, making it one of the most significant contributors to plastic pollution. HDPE offers good resistance to alkalis, dilute acids, and greases. Because of its exceptional strength, it is widely used to produce milk containers, lubricating oil containers, shampoo bottles, detergent bottles, recycling bins, and grocery bags, among other things. HDPE wastes have a high potential for use as a feedstock for pyrolysis and can be recycled many times^4^. Ning Liu et al.^25,26^ recovered energy from the waste polymers and used it to solar energy applications. It is not only environmentally friendly, but it also offers a green way of creating porous carbons for a wide range of applications by converting low-cost waste polymers into high-value-added energy utilization.

The catalytic process is characterized by the use of a catalyst to effect conversion. The pyrolysis of plastic waste involves several process factors, such as temperature, heating rate, catalyst usage, particle size, retention time, moisture content, and feedstock composition, amongst other things. Compared to thermal pyrolysis, the method showed a high likelihood of the transformation of synthetic waste into oil and improved quality at lower reaction durations and temperatures than was previously thought. These variables can reduce energy consumption while simultaneously increasing the output of the whole pyrolysis process. In the pyrolysis process, thermal degradation occurs while the material is held under a vacuum. According to the manufacturer, the catalytic pyrolysis transformation of the HDPE polymers was carried out in a pyrolysis reactor. The shredded plastic trash is put into a muffle furnace that can be operate continuously at 600 °C. A digital controller which monitors and adjusts the temperature via the thermocouple. The catalytic pyrolysis reactor included with a vacuum pump to aid the conversion. The catalyst that is employed in this procedure will avoid the formation of any dioxins. Depending on the type of plastic materials, the reaction occurs at a specified temperature and time. Sixty minutes of response time was required for HDPE testing, and at 450 °C, HDPE was converted to pyrolysis oil. According to the results, the oil output for HDPE is 50% weight of pyrolysis oil with 25% wax formation and 25% gas, and coke formation is observed.

Many researchers are investigating the different additives that may improve the performance of alternative fuel produced from recycled plastics. Table 1 tabulated the waste plastic fuel investigations and their performance outcome with oxygenated additives. With its renewable bio-resources and oxygenated properties, ethanol is an attractive alternative fuel for diesel engines. These oxygenates are often used in engines due to their higher volatility and latent heating properties. Many studies^27,28^ have focused on optimizing diesel fuel, biodiesel, and alcohol mixes as alternate fuels in CI engines. However, there are significant drawbacks, including decreased heating value, phase separation, pour point, and hazardous storage and transit circumstances for ternary blends. Ethanol can be blended with diesel as a engine fuel, which has many favourable properties, including higher oxygen content, low viscosity, less sulfur content, high hydrogen-to-carbon ratio, and a high rate of evaporative cooling. Ethanol has a lower viscosity than pure diesel, ensuing in better atomization of fuel injected into cylinders and improved mixing with air when combined with diesel^29,30^. Additionally, since ethanol has a high latent heat of evaporation, blending it with diesel fuel may increase volume efficiency via the evaporative cooling effect of the ethanol during the intake and compression strokes.

**Table 1:** Waste plastic fuel blends and their performance with oxygenated additives.

Authors and Year	Blend	BTE	BSFC	CO	HC	NOx	Smoke	Additives/Methodology
Gnanamoorthi and Murugan (2019)^18^	50D50W 50D40W10DEE 50D40W10MEA	↑	↓	↓	↓	↓	↓	MEA DEE
Bridjesh et al. (2019)^8^	50D50W 50D40W10MEA 50D40W10DEE	↑	↓	↓	↓	↓	↓	MEA DEE
Das et al. (2020)^16^	D80W10E10 D70W15E15 D60W20E20	↑	↓	↓	↓	↓	–	Ethanol/Varied the compression ratio
Sudershan et al. (2021)^19^	70 PPO + 18D + 12E 70 PPO + 24D + 6E 70 PPO + 28D + 2E	↓	↓	↓	↓	↓	↓	Ethanol/Varied the Injection timing, Injection Pressure
Gadwal et al. (2019)^20^	70PPO + 15D + 15E 80PPO + 10D + 10E 90PPO + 5D + 5E 90PPO + 0D + 10E	↓	↑	↑	↑	↑	↑	Ethanol/Varied the Injection timing, Injection Pressure
Selvam et al. (2021)^21^	W5DEE5 W10DEE5 W15DEE5 W20DEE5	↑	↓	↓	↑	↓	–	DEE
Kumar and Puli (2018)^22^	25PPO 25PPO5E 25PPO5M	↑	↓	↓	↑	↓	–	Tested at Petrol Engine with ethanol blend
Ravi and Karthikeyan (2019)^15^	D75-WPO 20-P05 D70-WPO 20-P10 D765-WPO 20-P15	↑	↓	↓	↓	↓	↓	Propanol
Govinda Rao et al. (2018)^23^	P90D10 P90D5E5 P80D10E10	↑	↓	↓	↓	↑	↓	Varied Compression Ratio and Injection pressure
Dillikannan et al. (2019)^24^	D 50-W 40-H 10 D 50-W 30-H 20 D 50-W 20-H 30	↓	↑	↓	↑	↓	↓	n-Hexanol

*BSFC* brake specific fuel consumption, *BTE* brake thermal efficiency, *CO* carbon monoxide, *DEE* diethyl ether, *E* ethanol, *EEA* ethoxy ethyl acetate, *H* n-Hexanol, *HC* hydrocarbons, *M* methanol, *MEA* methoxy ethyl acetate, *NO*x nitrogen oxides, *P* plastic oil, *ppm* parts per million, *PPO* plastic pyrolysis oil, *W*, *WPO* waste plastic oil.

↑: Increased; ↓: Decreased.

Utilize of oxygenated fuels seems to be a viable option for lowering emissions from diesel engines, both current and future. The term "oxygenated fuel" refers to a chemical substance that contains oxygen. It is used to increase fuel combustion efficiency and reduce the amount of air pollutants. Efforts have been made by Rao et al.^31^ to improve performance while also lowering emissions at the same time by altering the diesel with oxygenated additives. In order to create ternary blends, nitromethane and 2-Ethoxyethyl Acetate (EEA) were added to diesel fuel in various amounts. 2-Butoxy Ethanol and 2-Ethoxy Ethyl Acetate were studied by Srinivasan and Devaradjane^32^. When the oxygen concentration is raised from 5 to 15%, it reduces smoke, CO, and HC emissions while increasing Nitrogen Oxides (NOx) emissions. On the other hand, Deepanraj et al.^33^ studied the diesel engine’s characteristics running on EEA blends. The effects of several fuel blends, including 5%, 10%, and 15% EEA were studied on a DIDE. The EEA blended fuels improved engine performance and reduced emissions substantially when tested under various load situations.

The primary emphasis is on using waste plastic fuel derived from HDPE as a possible alternative fuel for diesel-powered engines. On reviewing the literature sources, it was learned that very little study was done on fuel derived from high-density polyethylene in the diesel engine when combined with ethanol and ethoxy ethyl acetate. Phase separation can be avoided by combining an additive such as EEA with waste plastic oil and a diesel mixture. The molecular compatibility and bonding additives operate as a bridging agent, resulting in a homogeneous blend. Many researchers, as stated in Table 1, tried various oxygenated additives like alcohols and ethyl additives with waste plastic fuel. The combination of two oxygenated additives with waste plastic fuel was not carried out in diesel engines. The blend ratio of additives was adopted between 5 and 15% with WPF in many of the investigations^15,16,19,20,22,23^. Hence, the addition of additives is preserved at 10% on a volume basis, which is considered the optimum ratio. In this research, a combination of 10% ethanol and 10% ethoxy ethyl acetate was added with three incremental ratios of waste plastic fuel as a quaternary fuel to evaluate the emission characteristics and performance of a single-cylinder diesel engine.

In this research, waste plastic fuel obtained from the pyrolysis process were blended with diesel at different ratios of 20%, 30% and 40% on a volume basis, along with oxygenated additives. The quaternary fuel named as WEE, formed by the blends of Waste Plastic Fuel, 10% ethanol and 10% ethoxy ethyl acetate on volume basis mix with pure diesel. The quaternary fuel blends prepared as WEE20 (60% diesel + 20% WPF + 10% Ethanol + 10% ethoxy ethyl acetate), WEE30 (50% diesel + 30% WPF + 10% Ethanol + 10% ethoxy ethyl acetate), and WEE40 (40% diesel + 40% WPF + 10% Ethanol + 10% ethoxy ethyl acetate).

### Experimental details

**Figure 1:**
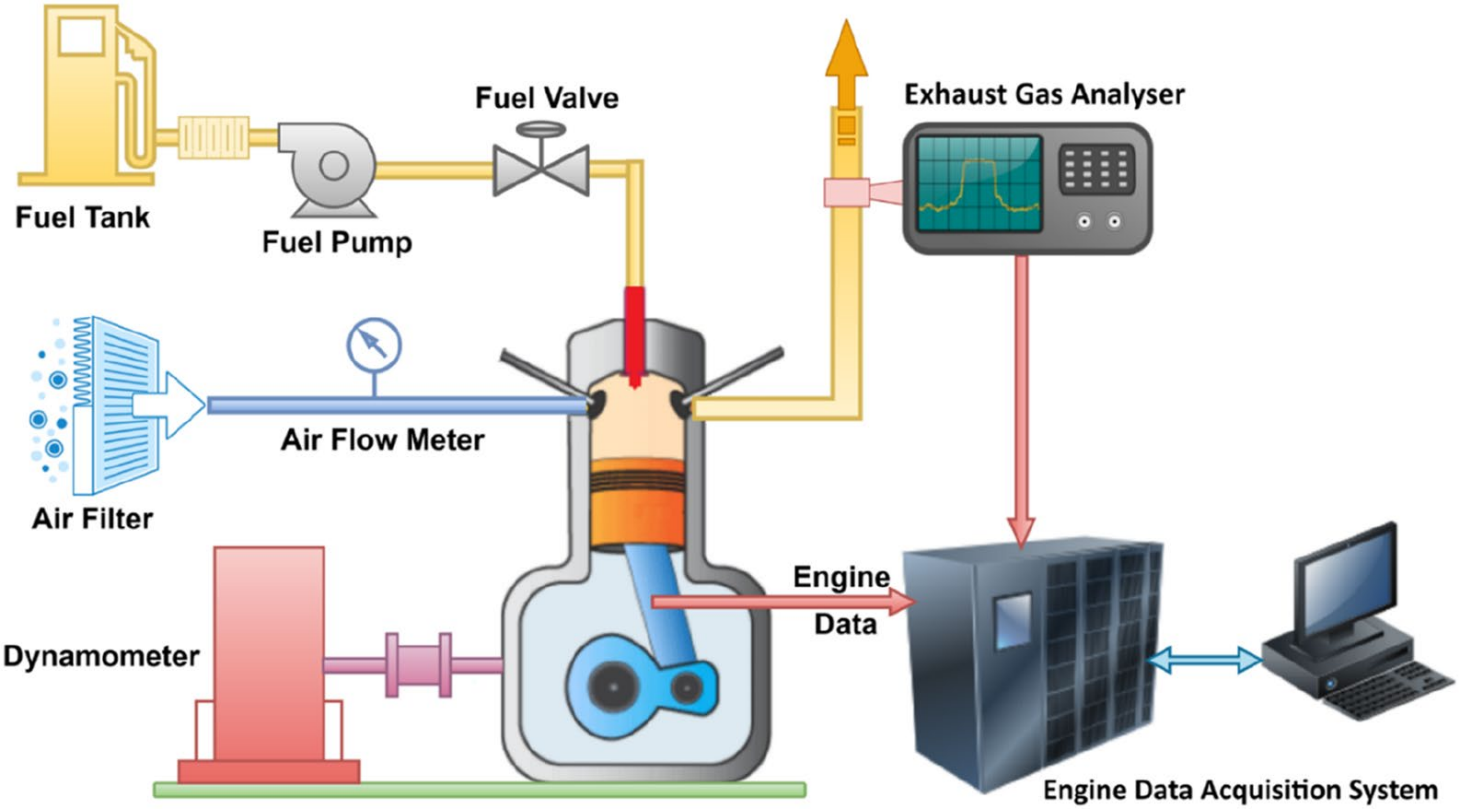
Schematic engine setup.

The engine study might be carried out with the aid of a water-cooled single-cylinder DIDE with a 4.2 kW output. The test engine started using the hand-cranking technique. The diesel engine was linked to an eddy current dynamometer to measure its performance. Using a dynamometer may manually load the engine from zero to maximum load in increments ranging from up to 100%, depending on power generated. The test engine runs at 1500 rpm with 17:1 compression ratio and operated under standard test condition. The experiment was conducted at injection timing of 21° bTDC (before top dead centre) and with injection pressure 210 bar. Exhaust pollution from the engine was studied using an AVL di gas analyzer and smoke metre. The AVL programme was used to assess the engine's stability and pollution levels. The experimentation engine setup is displayed in Fig. 1. The properties of Waste Plastic fuel, ethanol and ethoxy ethyl acetate were tabulated in Table 2. The fuel properties of WPF have several vital aspects similar to that of diesel fuel. However, the combustion and emission properties of the prepared fuel are to be determined, and instruments details are tabulated in Table 3.

**Table 2:** Fuels properties utilized for quaternary fuel combination.

Properties	Diesel	WPF	Ethanol	EEA
Chemical formula	C_10_H_22_	(C_2_H_4_)_n_	C_2_H_5_OH	C_6_H_12_O_3_
Density at 20 °C (kg/m^3^)	829	799	789	975
Calorific valve (MJ/kg)	45.3	42.8	26.8	23.6
Cetane number	52	65	8	61
Viscosity at 40 °C (cSt)	2.52	9.24	0.8	1.32
Auto ignition temperature (°C)	289	258	363	379
Oxygen content wt.%	0	4.5	35	17

**Table 3:** Experimentation instruments details.

Measurement	Range	Accuracy	Percentage of uncertainty	Instrument
Load	–	+ 0.1 to − 0.1 kg	± 0.50	Load cell
Speed	0–10,000 rpm	± 10 rpm	± 0.10	Digital tachometer
Fuel quantity	0–50 cm^3^	± 0.1 cm^3^	± 0.50	Burette Measurement
Hydro Carbon	0–15%	± 0.03%	± 0.15	AVL exhaust gas analyser, NDIR technique
Carbon Monoxide	0–20,000 ppm	± 10 ppm	± 0.30	AVL exhaust gas analyser, NDIR technique
Nitrogen Oxides	0–5000 ppm	± 10 ppm	± 0.25	AVL exhaust gas analyser, NDIR technique
Smoke	0–100%	± 1%	± 1	AVL smoke meter
Exhaust temperature	0–1300 °C	± 1 °C	± 0.15	Thermocouple

## Results and discussion

An investigation on the performance and emission characteristics of waste plastic fuel in a diesel engine has been conducted. Using diesel as the baseline fuel and quaternary fuel blends made up of 10% ethanol and 10% ethoxy ethyl acetate on a volume basis, blended with various ratios of 20%, 30%, and 40% of WPF, the test was carried out on a diesel engine. The assessment was carried out on an unaltered single-cylinder diesel engine operating at a 25% incremental load from 0 to 100% of its maximum capacity. Hydrocarbons (HC), nitrogen oxides (NOx), carbon monoxide (CO), and smoke are among the exhaust gases produced by engines that are being analyzed.

### Performance characteristics

**Figure 2:**
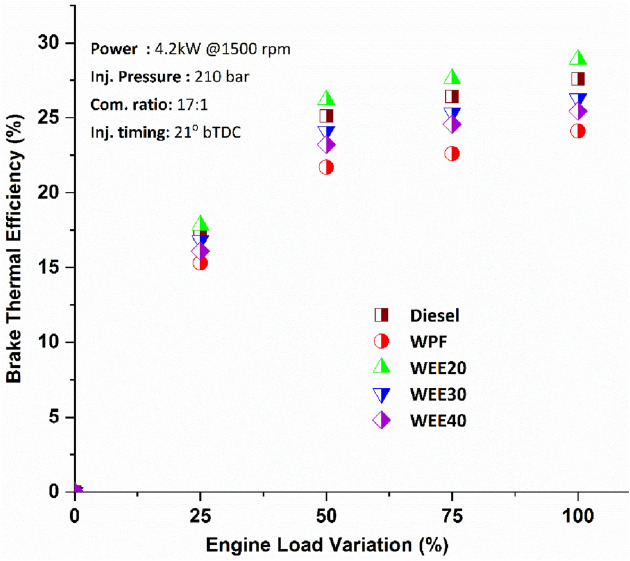
Performance of Brake thermal efficiency on engine loads.

Engine performance was observed with the brake thermal efficiency (Fig. 2) of 27.61%, 24.12%, 28.92%, 26.26%, and 25.45% at full load for the Diesel, WPF, and quaternary fuel blends of WPF with oxygenates additives. The brake thermal efficiency ranges from 17.84 to 28.92%, 16.81 to 26.26%, and 16.1 to 25.45% at various loads with oxygenates additives. When evaluated at maximum load, the BTE of WEE20 was about 4.74% greater than that of diesel and almost 20% higher than that of WPF. Improved BTE outcomes of 22%, 12%, and 8% have been observed with quaternary blends WEE20, WEE30, and WEE40 when compared to waste plastic fuel under various loading. The utilization of waste plastic fuel mixes resulted in a higher BTE being achieved.

The addition of ethanol and ethoxy ethyl acetate to waste plastic fuel positively affected fuel burning. An increase in oxygen content due to the presence of oxygen molecules in additives may be responsible for this, which would result in more efficient burning due to the presence of oxygen molecules in the additives^34^. Because less energy is lost in the combustion process due to a lower exhaust temperature, higher engine performance may be achieved. Plastic fuel contains a larger concentration of aromatic compounds, it takes a lot of energy to break the polymerization chain of plastic fuel. Fuel injection issues and poor spray quality may also be blamed on WPF's higher viscosity for its worse thermal efficiency under different load circumstances compared to other evaluated fuels^3^.

**Figure 3:**
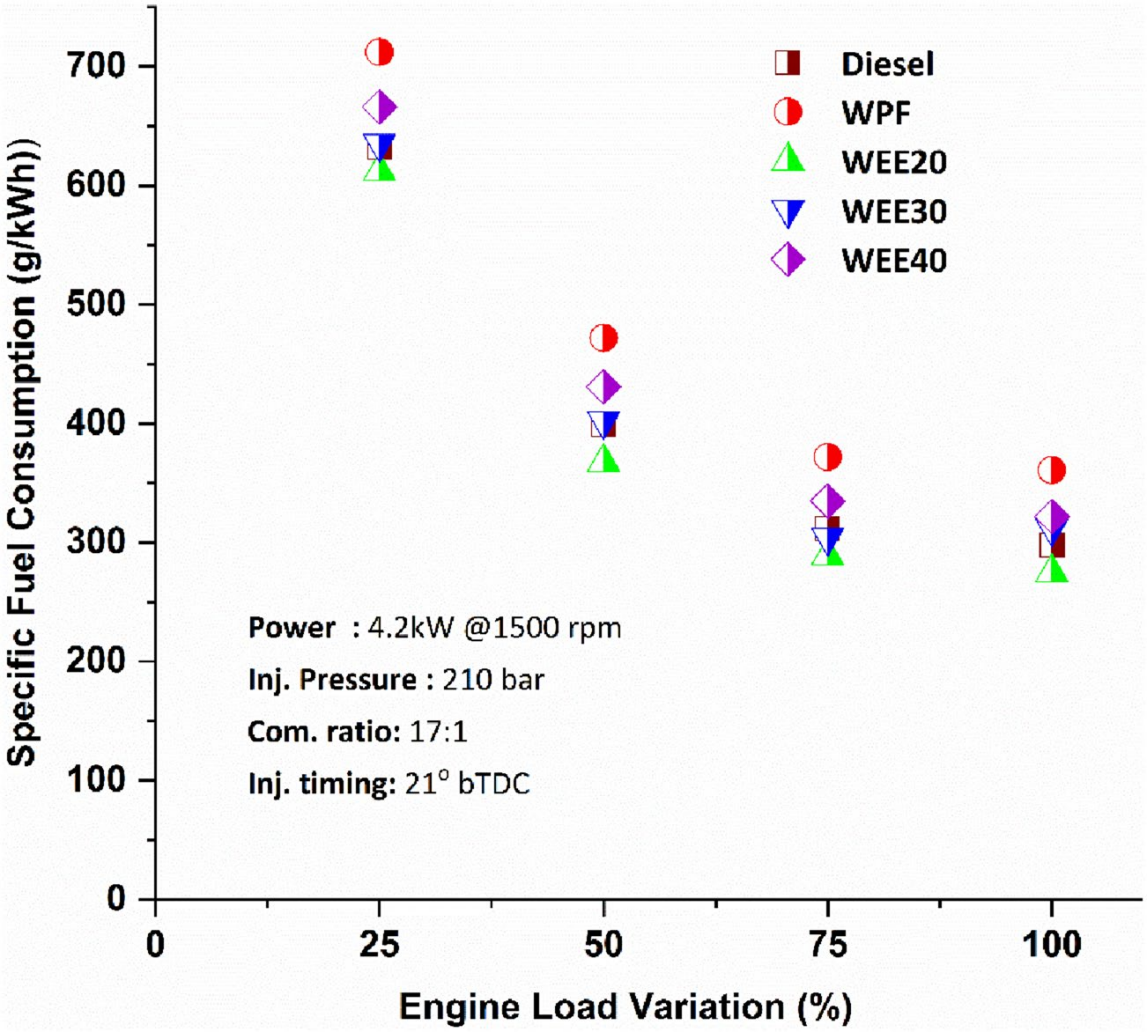
Performance of Specific fuel consumption on engine loads.

Specific fuel consumption of each engine are unique and vary depending on the engine's speed and load. The highest efficiency of a reciprocating engine is achieved only when the engine receives unthrottled air and when the engine is moving close to its torque peak. Figure 3 depicts the change in specific fuel consumption as a function of ternary blends under various loading scenarios. Specific fuel consumption decreases for WEE20 about 3.16% to 7.77% at various load conditions with diesel. There was a considerable reduction in fuel usage ranging from 14.1% to 23.8% at various load circumstances compared to the WPF. An increase in efficiency is obtained by the use of highly oxygenated conditions that require less fuel to provide the same amount of power output. More importantly, the calorific value of the blends influences engine output.

The waste plastic fuel has low calorific value than the pure diesel. It has an impact on the development of the fuel spray formation, which results in partial combustion. This results in the reduction in thermal efficiency, and the higher specific fuel consumption. It was observed that 275 g/kW-hr, 312 g/kWhr and 322 g/kWhr for quaternary blends WEE20, WEE30, and WEE40 respectively when compared to waste plastic fuel, which recorded 361 g/kWhr at maximum load. Due to the lower calorific value and higher viscosity of WPF and its blends, the fuel usage is greater than that of diesel operation^19^. The lower heating valve of mixed additives resulted in higher fuel consumption to provide the same amount of power, increased ignition delay owing to the low cetane number, and reduced combustion temperature due to ethanol's quenching action^18^.

**Figure 4:**
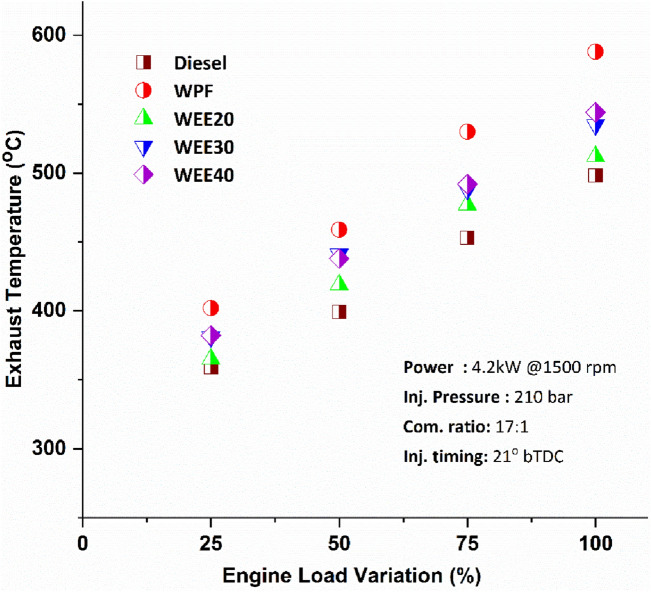
Variation of Exhaust gas temperature on engine loads.

The exhaust gas temperature in diesel engines varies significantly depending on the quantity of heat released in the engine chamber during the combustion cycle. Exhaust Gas Temperature (EGT) can also provide a strong overview of performance, air–fuel ratio, combustion heat, and available oxygen levels. The combustion temperature influences the EGT, contributing to an increase in the exhaust temperature with increasing load^35^. The WPF and quaternary blends’ EGT observed was more than the diesel at all loads conditions (Fig. 4). WEE20 recorded a 5.3% increased EGT, and other blends also observed higher temperature around 9–10% over the diesel. WPF shows incomplete combustion due to the higher viscosity and lower volatility, resulting in higher EGT. The higher EGT has been observed because some gases undergo combustion at the end of expansion stroke^36^. The quaternary blends contain more oxygen content which promotes combustion resulting in higher EGT in all load conditions.

### Emission characteristics

**Figure 5:**
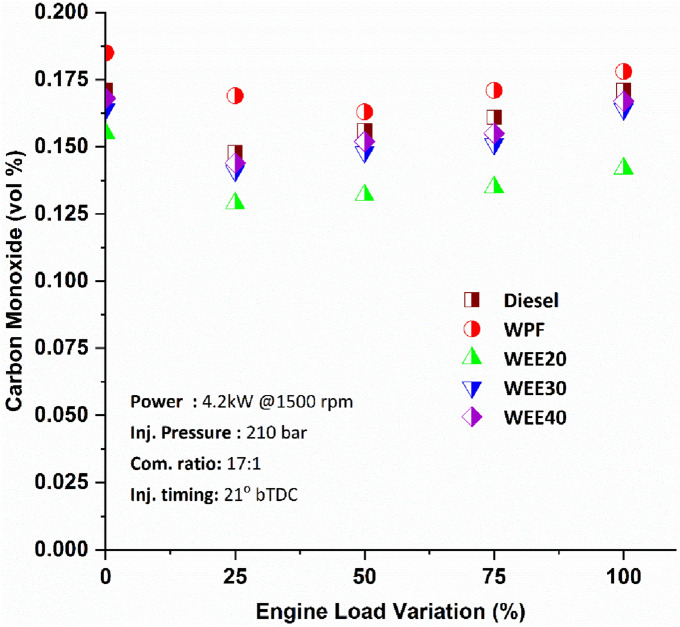
Variation of Carbon monoxide emissions on engine loads.

Carbon monoxide emissions are mostly produced by inadequate oxygen availability or poor oxygen usage in the combustion process. When compared to diesel, quaternary blends emit much less carbon monoxide at all loads. Figure 5 shows that carbon monoxide emissions drop gradually from lower load to half load and then rise till reaching full load for all of the mixes presented. Testing at maximum loads found CO emissions from WEE20 is to be 13.41% lesser than diesel and about 20.22% lesser than from WPF. Compared to diesel, the quaternary blends WEE20, WEE30, and WEE40 showed significant CO reductions of 13.41%, 6.21%, and 3.73%, respectively, when the loading was varied. Comparing quaternary blends to WPF, it is shown that there is a 9% to 23.6% decrease in carbon dioxide emissions. The quaternary blends have a higher concentration of oxygen, which allows for greater effective burning of the fuel. It results in a reduction in CO emissions as more fuel particles get oxidized. Addition of alcohols with low cetane numbers increases the ignition delay time during combustion. As a result of the OH group's effect, the majority of the alcohols undergo H-abstraction by OH radicals from the carbon position, as had previously been reported^37^. As a result of the ignition delay time, more fuel–air mixing occurs, resulting in improved combustion and CO reduction. A negative impact on carbon monoxide emissions was shown by waste plastic oil, whose increased viscosity results in inefficient atomization of fuel mixes, ultimately leading to increased carbon monoxide emissions^34^.

**Figure 6:**
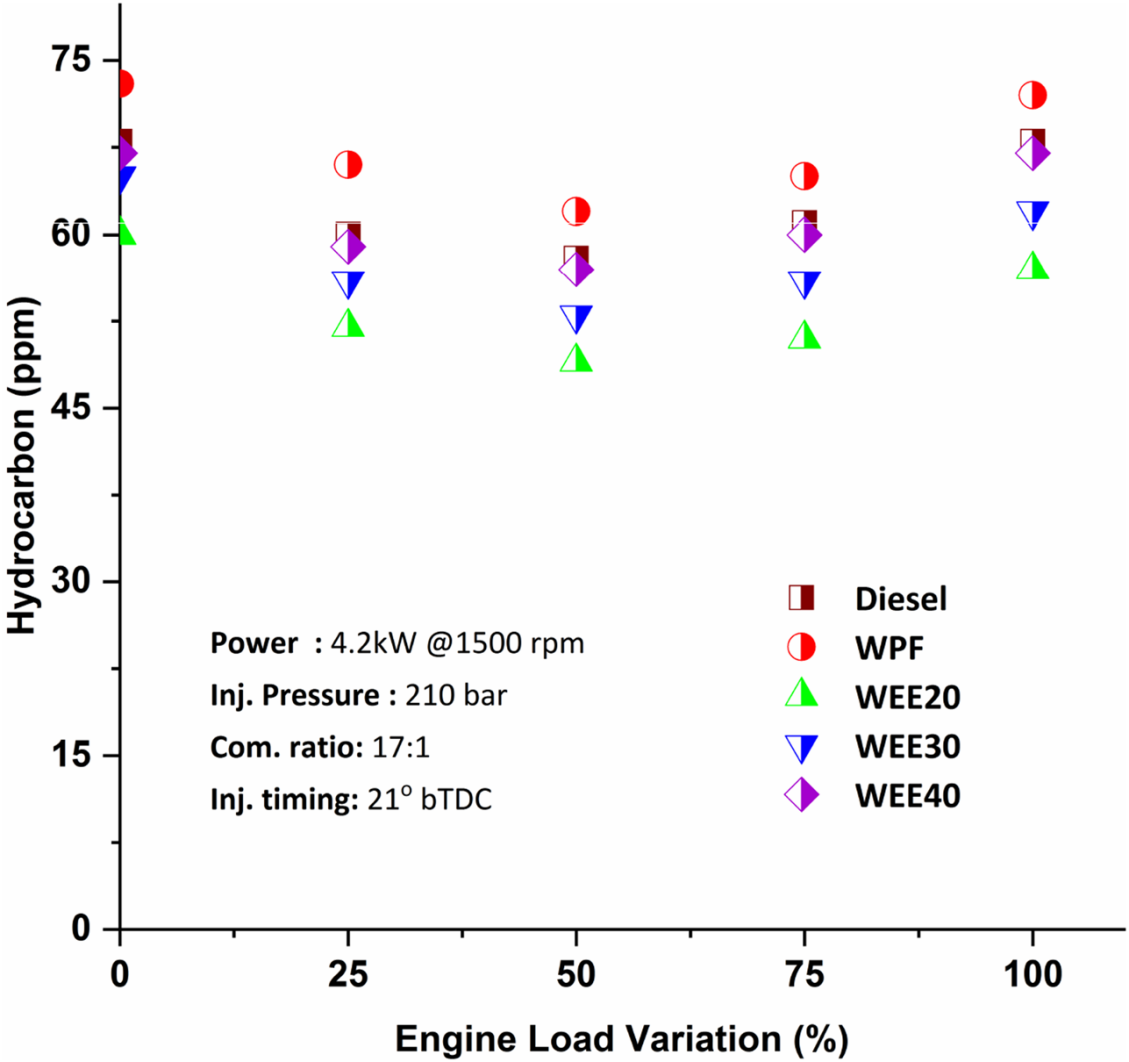
Variation of hydrocarbon emissions on engine loads.

Hydrocarbon emissions are mostly generated by inadequate mixing of fuel and air particles inside the combustion process, as well as by incomplete combustion of the fuel itself. When comparing diesel with quaternary blends of WEE20, WEE30, and WEE40 (Fig. 6), the hydrocarbon emission decreases about 11.76 to 16.39%, 4.41 to 8.82%, and 1.47 to 1.72% at various load conditions respectively. Due to the higher oxygen concentration in the fuel blends and appropriate mixing of the fuel and air takes occurs within the combustion chamber when the fuel is burned. The amount of fuel particles burnt in the combustion chamber is thus greater than diesel. The hydrocarbon emissions of WEE20 were about 16% less than diesel and 21.5% less WPF at maximum load. Low engine speed and low fuel injection pressure are present during idle conditions, resulting in slightly rich combustion conditions required for the combustion's stability. When ethanol is mixed with diesel, the reduction of partial fuel-rich areas caused by the effect of oxygen and the improved atomization caused by the lower viscosity of the injected fuel are the main reasons for reducing HC emissions. Mani et al.^38^ found that the HC was 15% greater at maximum load when comparing plastic fuel to diesel. Because of unsaturated aromatic combinations in waste plastic, it has an imperishable character, resulting in a rise in hydrocarbon emissions^36^. The low cetane number of WPO and its lower auto-ignition characteristics contribute to the enhancement of the quenching effect in the leaner mixture region of the cylinder, which in turn contributes to the rise in the quantity of hydrocarbons emitted.

**Figure 7:**
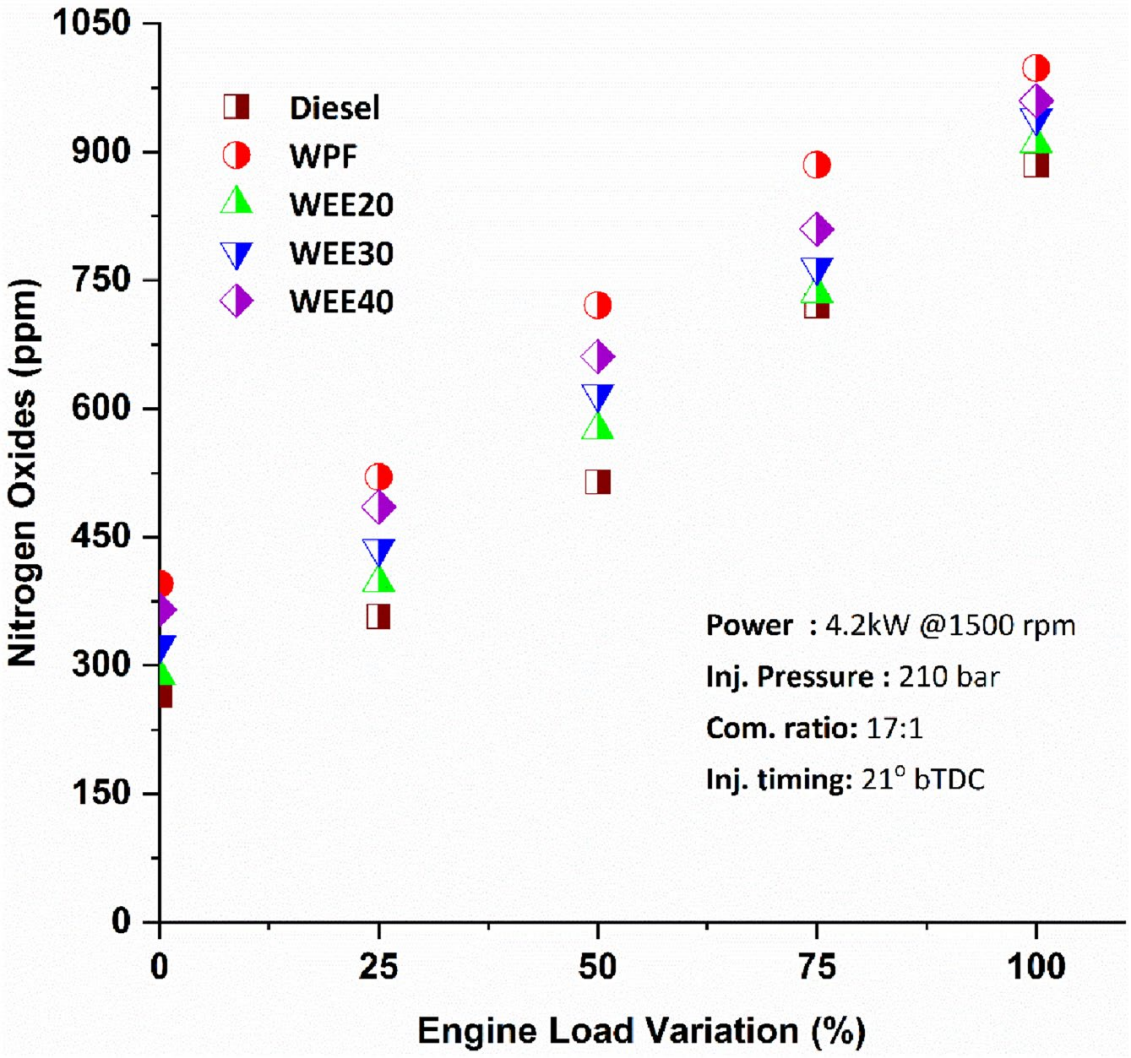
Variation of nitrogen oxide emissions on engine loads.

In diesel engines, NOx is produced primarily via the thermal mechanism and, to a lesser degree, through the prompt mechanism. At elevated temperatures, the thermal process results in an exponential rise in NOx levels. Nitrogen oxide emissions increased by 12.06%, 22.13%, and 35.85%, respectively, when quaternary blends WEE20, WEE30, and WEE40 were compared to diesel fuel at different loadings (Fig. 7). Increased NOx emissions from quaternary blends are caused mainly by increased fuel blend's combustion temperature. Completion of combustion occurs as a result of the increased oxygen concentration in mixed fuel. As a consequence, the combustion temperature rises, increasing the amount of NOx emitted. When oxygenates are added to diesel fuel, the fuel becomes more oxygenated. As a result, the combustion chamber was running lean. Oxygenated fuel provides the additional oxygen needed to oxidise the nitrogen. Oxygenated fuel's NOx emissions increase as a consequence.

Mani et al.^38^ found that, the NOx emissions were 25% higher for plastic fuel was compared to diesel at full load. As shown in Fig. 6, the NOx emission levels of all tested fuels are rising. Excess oxygen concentration has the most significant impact on the generation of NOx emissions in the cylinder. Nitrogen chains break down and disintegrate when exposed to high temperatures. Following that, these nitrogen bonds interact with the oxygen molecules trapped inside the cylinder's monotonic configuration. Emissions from waste plastic fuel were found to be between 12 and 50% more than those from diesel. WPF has more carbon-number compounds, which decreases surplus air availability, which leads to increased temperatures, which results in a rise in NOx.

**Figure 8:**
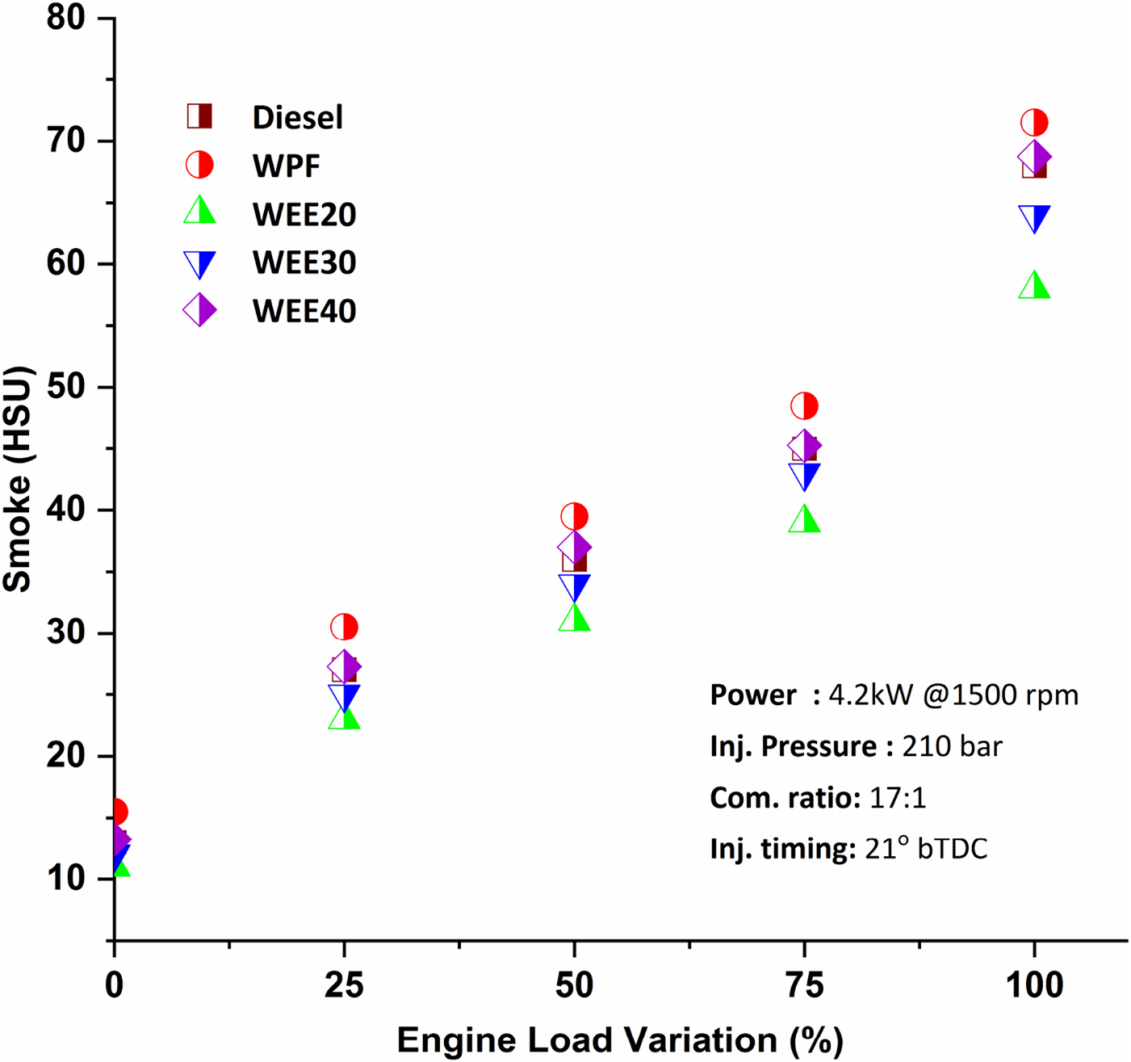
Variation of smoke emissions on engine loads.

The engine exhaust is a visual indication of the engine's combustion process. Smoke is produced when fuel is burnt inefficiently, resulting in unburned carbon particles. Smoke is formed in engines during the diffusion combustion stage. All the fuel atomized droplets are split into elementary carbon atoms and are subsequently oxidized in the combustion zone. Smoke emissions also occur in the combustion-rich zone due to a shortage of air, a more excellent carbon-to-hydrogen ratio, a higher viscosity of the fuel, insufficient atomization, and an excessive fuel buildup inside the combustion chamber. According to Fig. 8, compared to diesel, the amount of smoke generated by WEE20 and WEE30 blends decreases by between 8 and 9.38% and 4.44 to 7.69%, respectively. WEE40, on the other hand, reported a modest increase of 2% in smoke.

In comparison to diesel fuel, quaternary blends emit less smoke. It is primarily due to the synergistic impact of a higher cetane number and the presence of oxygen in fuel mixes. The cetane number indicates the quality of the ignition: the higher the cetane number, the more flammable the fuel. As the cetane number rises, the fuel's ignition quality improves as well. When the ignition quality of the fuel improves, the fuel burns more efficiently within the combustion chamber. As a result, the engine produces fewer unburned carbon particles. Additionally, the oxygen in the fuel aids in fuel combustion, reducing smoke output. Ravikumar and Senthilkumar^39^ found an 8.6% to 21.28% reduction in smoke in the coated engine than a standard diesel engine. Compared to diesel, waste plastic fuel produced an 18.8% to 39% greater amount of smoke. WPF has a more significant proportion of aromatic components, which results in incorrect fuel mixture development and spray production, resulting in incomplete combustion and significant smoke emission^13^. Another reason for incomplete combustion is that WPF has a higher viscosity and is less volatile^12^.

### Environmental impact of waste plastics and ethanol

Plastic goods are ubiquitous in the workplace and home surroundings of humans. Plastic pollution has the potential to harm and pollute the terrestrial ecosystem. Additionally, plastic contributes to global warming. Plastic remains in the environment for an extended period, endangering animals and spreading poisons. Each year, plastics kill millions of animals, from birds to sea livings (Okunola et al.^40^). On the other hand, diesel emissions cause cancer, cardiovascular and respiratory diseases, air, water, soil pollution, soiling, reduced visibility, and global climate change. Carbon monoxide affects the number of greenhouse gases related to climate change and global warming. CO causes acute poisoning when combined with hemoglobin to form carboxy-hemoglobin (COHB), preventing adequate oxygen transport from the lungs to human tissues. As a COVID-19 symptom, excessive CO concentrations impair proper respiratory system function (Adefeso et al.^41^). Hydrocarbons are very harmful to humans. Intake of hydrocarbons affects the immune system, hepatic, respiratory, reproductive, circulatory, and renal systems. Human effluents contaminated by hydrocarbons also cause cancer and hormonal issues that may disrupt development and reproduction (Srivastava et al.^42^).

Because ethanol is water-soluble, biodegradable, and easily evaporated, it may provide some safety benefits over fossil fuels. Ethanol fuel is the most cost-effective energy source since it can be produced in almost any country. Ethanol is a type of fuel derived from corn and other plants. There are many various forms of ethanol, but the most common is E10, and the blend ratio varies from 10 to 15% over the world. Many nations, like Brazil and the United States, allow for the use of a high-level ethanol fuel blend containing 50–85 percent ethanol^43^. Because ethanol is easily produced, it is less expensive than fossil fuel. The primary by-products of ethanol fuel combustion are carbon dioxide and water. In terms of pollution, the carbon dioxide emitted has little impact. The burning of ethanol made from biomass such as corn and sugarcane, on the other hand, is regarded to be "atmospheric carbon neutral". This is due to the fact that when biomass grows, it absorbs CO_2_, which may offset the CO_2_ emitted when ethanol is burned^44^.

The linear economy focuses on the feedstock, the manufacturing process, and the delivery of the final product. The product's afterlife was never given the consideration it deserved. The condition of the product after it has reached the end of its useful service life has been overlooked. Plastic products might be disposed of in landfills or incinerated as an alternative to recycling. Gong et al.^45^ and Zhang et al.^46^ have developed alternative solutions for the energy recovery of waste polymers into electrochemical storage and steam evaporation systems, which are the ideal polymer waste recovery methods.

A refinery using recycled plastic decreases oil consumption, lowering capital expenditure on exploration and increasing oil reserves. Plastic production uses around 8% of the world's oil, with roughly half of that usage going to the creation of monomers and the other half going to the production of energy. Physical and chemical treatments must be widely implemented in order to be commercially sustainable. The proposed approach by Palos et al.^47^ suggests establishing a new waste management business network. The oil industry would gain from the business network's commitment to sustainable development.

This research focuses on recovering energy from plastic waste and utilizing bio-cropped ethanol to achieve the circular economy approach as a potential fuel for transportation vehicles. Because of the high energy density of hydrocarbons found in plastic, they are excellent fuel sources. The quantity of recyclables that can be recycled without degrading the strength is one of the issues facing the circular economy. When it comes to offering a cost-effective end-of-life solution, plastic pyrolysis and combustion are viable options since they allow for the production of value-added goods while also reducing environmental impact. Recycling and reusing discarded plastic has the potential to save and recover a great deal of energy. Similarly, in the period of 2020–2021, India's net petroleum imports were 185 Mt at $551 billion, the ethanol blending E20 programme that works may save the government $4 billion annually. Ethanol is also less polluting and cheaper than fossil fuels. E20 is a national need and a strategic demand due to abundant arable land, expanding food grain and sugarcane output, and the capacity to convert automobiles to ethanol mixed fuel. In two-wheelers, the CO emission drop was 50%, and in four-wheelers reduction up to 30%. Also, blends of ethanol and gasoline lower hydrocarbon emissions by 20%^48,49^.

The objective of this study is to determine the performance of waste plastic fuel generated from the pyrolysis of HDPE in a diesel engine. A quaternary fuel blend of WPF was developed to combat the high-value emissions of WPF during diesel engine performance. The blends included 10% ethanol and 10% ethoxy ethyl acetate as an oxygenated additive to reduce the harmful emissions. The outcome of the WPF blends results in better fuel economy of up to 20% better than diesel and a reduction in tailpipe emissions of around 13% of CO and 16% of HC compared to fossil fuel. Similarly, the ethanol blending programme in India will reap numerous benefits, including annual savings of Rs.30,000 crore in foreign exchange, energy security, lower carbon emissions, better air quality, self-reliance, the use of damaged foodgrains, an increase in farmer incomes, the creation of new jobs, and increased investment possibilities^48^. The usage of energy recovered WPF and oxygenated additives is possible to combat climate change by reducing greenhouse gas emissions from engines via better fuel efficiency, enhancing the country's energy needs and boosting the economy^48,49^.

## Conclusions

Waste plastic, which poses a significant problem in terms of disposal, may be converted into energy. This study investigates the possibility of recovering energy from waste plastics as a potential option to meet the circular economy as a fuel source. The goal of this research is to investigate the performance of waste plastic fuel created from the pyrolysis of HDPE in a diesel engine. A quaternary fuel blend including three distinct ratios of WPF was developed to combat the high-value emissions of WPF during diesel engine performance. The blends included 10% ethanol and 10% ethoxy ethyl acetate as an oxygenated additive to produce quaternary fuel blends. The following observations were made on the quaternary fuel blends on single-cylinder diesel engines, the brake thermal efficiency of WEE20 is 4.74% greater than diesel and almost 20% higher than that of WPF at maximum load. Improved BTE outcomes of 22%, 12%, and 8% have been observed with quaternary blends with WPF. Specific fuel consumption decreases for WEE20 about 7.77% with diesel and considerable reduction in fuel usage ranging from 14.1% to 23.8% at various load circumstances compared to the WPF. WEE20 recorded a 5.3% increased EGT, and other blends also observed higher temperature around 9–10% over the diesel. At maximum loads, CO emissions from WEE20 is 13.41% lesser than diesel and about 20.22% lesser than from WPF. The quaternary blends WEE20, WEE30, and WEE40 showed significant CO reductions of 13.41%, 6.21%, and 3.73%, respectively. The hydrocarbon emissions of WEE20 were recorded at maximum load, and it was about 16% lower than that of diesel and 21.5% lower than WPF. The hydrocarbon reported decreases by about 16.39%, 8.82%, and 1.72% with diesel when comparing quaternary blends. Nitrogen oxide emissions increased by 12.06%, 22.13%, and 35.85% on quaternary blends WEE20, WEE30, and WEE40, respectively, were compared to diesel fuel at different loadings. A decrease in smoke produced by WEE20 and WEE30 blends is between 8 and 9.38% and 4.44 to 7.69%, respectively. According to the results of this research, waste plastic fuel has the potential to be utilized as an alternative energy source for boilers, industrial engines, marine engines, and even locomotive diesel engines. Furthermore, energy recovered WPF and ethanol blends will help to energy demand, improve air quality, reduce carbon emissions, raise farmer revenue, create employment, and expand investment opportunities, thereby contributing to the country's economy.
